# Mathematical model for managing vector-borne pathogen outbreaks in chickens using impulsive vaccination and drug treatment

**DOI:** 10.1038/s41598-024-83510-4

**Published:** 2024-12-30

**Authors:** Kamonchat Trachoo, Din Prathumwan, Darunee Padasee, Supatcha Paopan, Inthira Chaiya

**Affiliations:** 1 Department of Mathematics, Faculty of Science, Mahasarakham University, Mahasarakham, 44150 Thailand; 2 Department of Mathematics, Faculty of Science, Khon Kaen University, Khon Kaen, 40002 Thailand

**Keywords:** Mathematical model, Periodic impulsive vaccination, Vector-borne pathogen, Epidemic model, Stability, Applied mathematics, Infectious diseases

## Abstract

In this paper, we propose an epidemic mathematical model with an impulsive vaccination strategy to predict outbreaks in chickens caused by vectors. The analysis of the model is divided into two parts: one considering impulsive vaccination and the other without it. We determine the basic reproduction number of disease transmission and analyze the stability conditions of the proposed model for both disease-free and endemic equilibria, addressing both local and global stability. The results reveal that the disease will die out when the basic reproduction number is less than one. Numerical simulations demonstrate that impulsive vaccination significantly reduces the number of exposed and infected chickens, leading to disease eradication in approximately 270 days, compared to over 360 days without impulsive vaccination. The existence and non-negativity of the model solutions are also investigated. The susceptible population is considered to be vaccinated. We find that the periodic solution of the disease-free equilibrium is locally asymptotically stable under specific conditions outlined in the proposed theorem. This highlights the effectiveness of impulsive vaccination strategies in controlling disease transmission.

## Introduction

Poultry farming is a global industry known for its low investment requirements and adaptability to various climates. However, inadequate health management in chicken farming exposes these birds to diseases, often transmitted by disease-carrying insects. Infected chickens may not exhibit clear symptoms or may show sudden signs such as pale blood, lethargy, reduced food intake, weakness, rapid breathing, diarrhea, and potential neurological symptoms. Common diseases in chickens transmitted by insects include Newcastle disease, Leucocytozoonosis, Pox, and Avian Malaria. Infected chickens can experience a high mortality rate, approximately 80%^1–3^, leading to a reduction in egg production from 10 to 40%. The fall in production can be rapid, and the drop in egg production usually lasts from 4 to 10 weeks^4^. These outcomes result in significant economic losses for poultry farmers. Therefore, disease control becomes crucial to mitigate economic losses associated with poultry farming.

Understanding and managing diseases in chickens is crucial for the global poultry industry facing shared challenges. Mathematical models offer valuable insights into disease transmission dynamics and control measures. There are many reseach investigated the disease transmission with vectors such as Fantaye^5^ proposed the mathematical model of cotton leaf curl virus (CLCuV) disease in cotton plants by adding vector population into the model. Diabete et al.^6^ proposed and analyzed the mathematical model of vector-borne disease involving human and mosquitoes. Fantaye et al.^7^ developed the mathematical model of skin sores (impetigo) disease and find the conditions for stability which involved the basic reproduction number. Fantaye and Birhanu^8^ proposed the mathematical model of corruption dynamics with optimal control. Liana and Swai^9^ studied the coccidiosis disease in chicken and proposed the mathematical model to express the disease transmission. Notably, various mathematical models have been proposed for disease control in chickens. In 2020, Ijeoma et al.^10^ demonstrated the efficacy of a five-compartmental model for Newcastle Disease, showcasing the impact of combined vaccination therapy and optimal vaccine efficacy on reducing infectious bird populations. Concurrently, Xie et al.^11^ introduced a robust numerical scheme exploring the stochastic influenza avian model, underscoring the impact of environmental noise on population dynamics. In 2021, Hugo et al.^12^ investigated mathematical control methods for infectious bursal disease in chicks, revealing the cost-effectiveness of combining chick vaccination and environmental sanitation for effective control with limited resources. Additionally, Muhumuza et al.^13^ proposed a stochastic model that, in 2022, emphasized disease extinction with infected mosquitoes and increased outbreak likelihood with infected chickens, highlighting the importance of transmission routes.

In this study, we propose a mathematical model capturing disease transmission in chickens, incorporating susceptible, exposed, infected, and quarantined chickens in the presence of disease-carrying insects. Our approach specifically utilizes impulsive vaccination to address the limitations of conventional vaccination methods by administering vaccines at strategic intervals, particularly upon the loss of immunity. Furthermore, we investigate the influence of impulsive vaccination on susceptible chickens. By exploring these scenarios, we aim to fill the existing gap in the literature and provide insights into more effective control measures. Both theoretical and numerical analyses of this model will be conducted.

The remainder of this paper is structured as follows: The model formulation is introduced in “Mathematical model” section, and the analysis of a non-vaccination reduced model is represented in “The dynamics of reduced model with no vaccination strategy” section. In “SEIHR model with impulsive vaccination” section, the impulsive vaccination model is analyzed. The numerical simulations are illustrated in “Numerical simulations” section, followed by the conclusions and discussion in “Conclusions and discussion” section.

## Mathematical model

To comprehensively explore strategies for controlling epidemics by using the impulsive vaccination, an innovative mathematical model for depicting disease transmission in chicken populations is presented. The proposed model intricately captures the dynamics of five distinct chicken classes these are the susceptible chicken class (*S*), the vaccinated chicken class (*V*), the latent or exposed chicken class (*E*), the infected chicken class (*I*), and the quarantined chicken class (*Q*). Additionally, the model includes two classes for disease-carrying insects: the susceptible vector class $(S_v)$ and the contaminated vector class $(C_v)$.

Within the susceptible class (*S*), representing chickens vulnerable to the disease, individuals are added to this class at the rate which defined by $\Lambda$. The model takes into account mortality rates associated with the presence of symptoms $(\delta )$. Furthermore, a natural death rate $(\mu )$ applies to all five chicken classes, while $(\mu _v)$ applies to all vectors, reflecting expected mortality in the absence of the virus.

The transmission rates $\beta _1$, $\beta _2$, and $\beta _3$ which use to express virus transmit between the population, quantifying the ease of virus spread. The transition rate which is denoted by $\phi$ can be described the rate of population to the infected class from the exposed class, where $1/\phi$ is the incubation period.

The transition rate that can express the chickens move from the infected compartment to the quarantined class is defined as $\rho$. Additionally, the model incorporates transitions of chickens from the vaccinated class back to the susceptible class upon losing immunity, can be determined by the rate $(\varepsilon )$.

The proposed model integrates the effect of impulsive vaccination on susceptible chickens. These chickens get vaccine, and booster shots may be administered when immunity wanes, the occurring rate can be represented by $\omega$, in a periodic process with a period of *T*. So, our model can be described by impulusive model as follows:

For $$t \ne nT,$$

1 $$\begin{aligned} \begin{aligned} \frac{dS}{dt}&= \Lambda -(\beta _1 I+\beta _2C_v)S -\mu S+\varepsilon V, \\ \frac{dV}{dt}&= -(\varepsilon +\mu )V, \\ \frac{dE}{dt}&= (\beta _1 I+\beta _2C_v)S-(\phi +\mu )E, \\ \frac{dI}{dt}&= \phi E-(\delta +\rho +\mu )I, \\ \frac{dQ}{dt}&= \rho I-\mu Q, \\ \frac{dS_v}{dt}&= \Lambda _v-\beta _3 S_vI -\mu _v S_v, \\ \frac{dC_v}{dt}&= \beta _3 S_vI-\mu _vC_v. \\ \end{aligned} \end{aligned}$$

For $$t = nT,$$

2 $$\begin{aligned} \begin{aligned} S(nT^+)&= (1-\omega )S(nT), \\ V(nT^+)&= \omega S(nT)+V(nT), \\ E(nT^+)&=E(nT), \\ I(nT^+)&=I(nT), \\ Q(nT^+)&= Q(nT), \\ S_v(nT^+)&= S_v(nT),\\ C_v(nT^+)&=C_v(nT), \end{aligned} \end{aligned}$$

where *T* represents the period between two subsequent vaccinations, where $n \in {\mathbb {Z}}_+$ and ${\mathbb {Z}}_+ = {1, 2, 3, \ldots }$. The parameter $\omega$ reflects the negative impact of vaccination on the susceptible class, considering its positive effect on the vaccinated class, with $0< \omega < 1.$

**Fig. 1:**
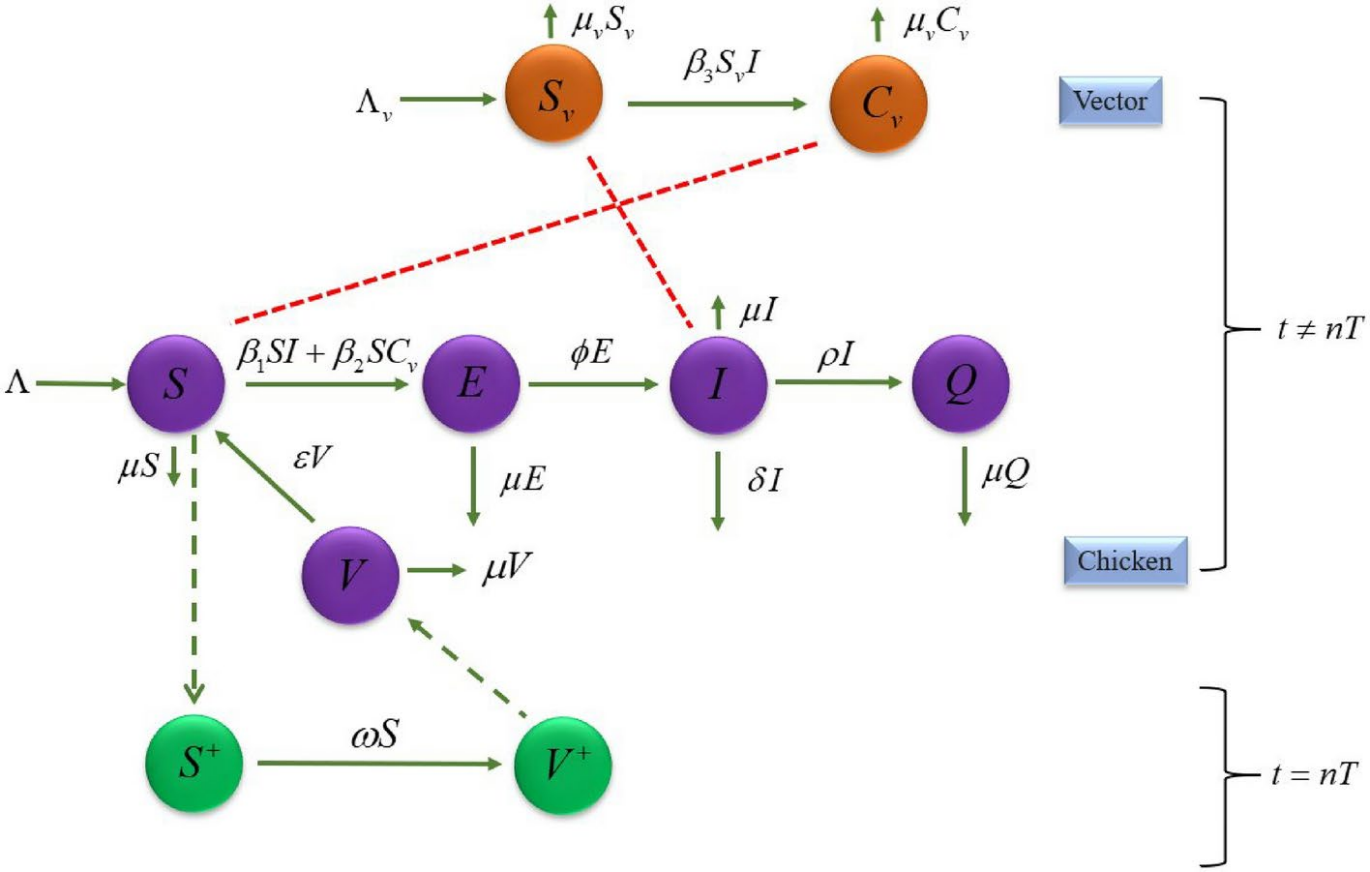
Flowchart of the impulsive vaccination model of the chicken disease.

A flowchart of the proposed impulsive vaccination model of the chicken disease by the system (1) and (2) is illustrated as in Fig. 1.

## The dynamics of reduced model with no vaccination strategy

Let us now scrutinize the simplification system in the absence of vaccination.

3 $$\begin{aligned} \begin{aligned} \frac{dS}{dt}&= \Lambda -(\beta _1 I+\beta _2C_v)S -\mu S, \\ \frac{dE}{dt}&= (\beta _1 I+\beta _2C_v)S-(\phi +\mu )E, \\ \frac{dI}{dt}&= \phi E-(\delta +\rho +\mu )I, \\ \frac{dQ}{dt}&= \rho I-\mu Q, \\ \frac{dS_v}{dt}&= \Lambda _v-\beta _3 S_vI -\mu _v S_v, \\ \frac{dC_v}{dt}&= \beta _3 S_vI-\mu _vC_v. \\ \end{aligned} \end{aligned}$$

### Existence of the solution

The existence of the solution is the important part to confirm that the proposed model has a solution. At the first step, we introduce the Lemma that tell us the conditions how to prove the existence of the solution.

#### Lemma 3.1

(Derrick and Groosman theorem^14^)* Let *$\Omega$* denote the region*

$$\begin{aligned} |t-t_0 | \le a\,,\, ||u-u_0|| \le 1,\, u =(u_1, u_2,\,\ldots ,\, u_n), u_0 = (u_{10}, u_{20},\,\ldots ,\, u_{n0}),\, \end{aligned}$$

*and suppose that f*(*t*, *u*)* satisfies the Lipchitz condition*

$$\begin{aligned} ||f(t,u_1)-f(t,u_2)||\le k||u_1-u_2|| \end{aligned}$$

*whenever the pairs* $(t,u_1)$* and *$(t,u_2)$* belong to *$\Omega$* where k is a positive constant. Then, there is a constant *$a \ge 0$* such that there exists a unique continuous vector solution of u*(*t*)* of the system in the interval *$t-t_0 \le a$.

*It is important to note that the condition is satisfied by the requirement that *$\partial f_i/\partial u_j\,\, \text {for} \,i,$ $j =1,2,3,\,\ldots ,n$* are continuous and bounded in *$\Omega$.

#### Theorem 3.2

*The solution of the model *(3)* with satisfying the initial conditions *$$S(0) \ge 0,E(0)\ge 0,\,$$$$I(0) \ge 0,$$ $$Q(0)\ge 0,\,$$$$S_v(0)\ge 0,C_v(0)\ge 0$$* exists and is unique in *$${\mathbb {R}} _{+}^{6}$$* for all *$$t \ge 0.$$

#### Proof

Define the functions to express the right-hand sides of the system (3) as follows:

$$\begin{aligned} \begin{aligned} f_1&= \Lambda -(\beta _1 I+\beta _2C_v)S -\mu S, \\ f_2&= (\beta _1 I+\beta _2C_v)S-(\phi +\mu )E, \\ f_3&= \phi E-(\delta +\rho +\mu )I, \\ f_4&= \rho I-\mu Q, \\ f_5&= \Lambda _v-\beta _3 S_vI -\mu _v S_v, \\ f_6&= \beta _3 S_vI-\mu _vC_v. \end{aligned} \end{aligned}$$

It is reasonable in showing that $\partial f_i /\partial u_i$ are continuous and $\displaystyle \left| \partial f_i /\partial u_i \right| < \infty$ for $i,j=1,2,\ldots ,6$, where $u_1=S, u_2=E, u_3=I, u_4=Q, u_5=S_v,$ and $u_6=C_v$. By Lemma 3.1, the system (3) has a unique solution. $\square$

### Invariant region

Let *N*(*t*) and $N_v(t)$ be the total number of chicken populations and vector populations at time *t*, respectively. It follows that

$$\begin{aligned} \frac{dN}{dt}&=\frac{dS}{dt}+\frac{dE}{dt}+\frac{dI}{dt}+\frac{dQ}{dt}\\&= \Lambda -\mu N-\delta I. \end{aligned}$$

Then,

$$\begin{aligned} \lim _{\sup t \rightarrow \infty } N\le \frac{\Lambda }{\mu }. \end{aligned}$$

Similarly,

$$\begin{aligned} \frac{dN_v}{dt}&=\frac{dS_v}{dt}+\frac{dC_v}{dt}\\&= \Lambda _v-\mu N_v. \end{aligned}$$

Then,

$$\begin{aligned} \lim _{\sup t \rightarrow \infty } N_v= \frac{\Lambda _v}{\mu _v}. \end{aligned}$$

As a result, the possible region for the system (3) is

$$\begin{aligned} \Omega =\left\{ (S,E,I,Q,S_v,C_v) \in {\mathbb {R}}^6:S\ge 0,E\ge 0,I\ge 0,Q\ge 0,S_v\ge 0,C_v\ge 0,N+N_v\le \frac{\Lambda }{\mu }+\frac{\Lambda _v}{\mu _v}\right\} . \end{aligned}$$

### Positivity of the solution

One of the most important thing in the epidemic mathematical model is the region of the solution. The solution must be positive for all time. The following theorem can express the positivity of the model’s solution.

#### Theorem 3.3

*The solution of the model *(3)* with satisfying the initial conditions *$S(0) \ge 0,E(0)\ge 0,I(0) \ge 0, \ Q(0)\ge 0,S_v(0)\ge 0,C_v(0)\ge 0$* is positive in *${\mathbb {R}}^{6}$* for all *$t \ge 0.$

#### Proof

Consider the positivity of *S*(*t*): from the first equation of the model system (3) which can be represented as $dS/dt=\Lambda -(\beta _1 I+\beta _2C_v)S -\mu S$. We consider the inequality: $dS/dt\ge -(\beta _1 I+\beta _2C_v +\mu )S$. By employing the separation of variables method and integration, the solution is obtained as follows: $S(t)\ge S(0)\text {exp}[- \int ^t_0 (\beta _1 I+\beta _2C_v+\mu ) \,dt].$ Consequently, we can confidently assert that $S(t) \ge 0.$

Futhermore, by employing the same process for all variables, we obtain the positiviy of all rest variables. As a result, the solutions of the model (3) always still non-negativity for all *t*. $\square$

### Stability analysis

#### Local stability of disease-free equilibrium (DFE)

To accomplish this, we set $E=I=C_v=0$ and all the equations in the system (3) to be zero. The DFE is

$$\begin{aligned} EE=(S^0,E^0,I^0,Q^0,S_v^0,C_v^0)=\left( \frac{\Lambda }{\mu },0,0,0,\frac{\Lambda _v}{\mu _v},0\right) , \end{aligned}$$

Following the work of van den Driessche^15^, we aim to determine the basic reproduction number, denoted by $R_0$. This value represents the average number of secondary infections caused by a single infectious individual in a completely susceptible population. To compute $R_0$, we will employ the well-established next-generation matrix method^16^. However, as our focus is on calculating the basic reproduction number from the perspective of new chickens becoming infected, we will not include new infections in the vector population in this calculation. This approach entails defining specific matrices, which we will elaborate on in the following :

$$\begin{aligned} f= \begin{pmatrix} \beta _1 SI+\beta _2SC_v\\ 0\\ 0 \end{pmatrix}, v= \begin{pmatrix} AE\\ -\phi E+BI\\ -\beta _3 S_vI+\mu _vC_v \end{pmatrix}. \end{aligned}$$

Then, matrices *F* and *V* are obtained by the Jacobian matrices of $f$ and $v$ as follows

$$\begin{aligned} F= \begin{pmatrix} 0 & \beta _1 S^0 & \beta _2S^0 \\ 0 & 0 & 0\\ 0& 0& 0 \end{pmatrix}, V= \begin{pmatrix} A & 0 & 0\\ -\phi & B& 0\\ 0& -\beta _3S_v^0& \mu _v \end{pmatrix}, \end{aligned}$$

respectively.

Additionally, we can write

$$\begin{aligned} FV^{-1}= \begin{pmatrix} \dfrac{\beta _1\phi S^0}{AB}+\dfrac{\beta _2\beta _3\phi S^0S_v^0}{\mu _vAB} & \dfrac{\beta _1 S^0}{B}+\dfrac{\beta _2\beta _3 S^0S_v^0}{\mu _vB}& \dfrac{\beta _2 S^0}{\mu _v} \\ 0 & 0 & 0\\ 0 & 0 & 0 \end{pmatrix}. \end{aligned}$$

The eigenvalues of $FV^{-1}$ are

$$\begin{aligned} \lambda _1 =\lambda _2 =0 \ \ \text {and}\ \ \lambda _3 =\frac{\phi S^0(\beta _2\beta _3S_v^0+\mu _v\beta _1) }{\mu _v AB}. \end{aligned}$$

Therefore, the basic reproduction number can be written as

$$\begin{aligned} R_0=\frac{\phi \beta _1 S^0}{AB}+\frac{\phi \beta _2\beta _3 S^0S_v^0 }{\mu _v AB} \end{aligned}$$

where $A=\phi +\mu$ and $B=\delta +\rho +\mu$. Each component of $R_0$ can be elucidated as follows:

$\beta _1 SI$ represents the rate of infection among susceptible chicken individuals per unit time for symptomatic cases. Thus, the rate of infection for one symptomatic chicken individual is $\beta _1 S$, and the average duration of symptomatic infection is $\frac{1}{B}$. Asymptomatic chicken individuals transition to symptomatic status at a rate of $\frac{\phi }{A}$ per unit of time. The first term of $R_0$, $\frac{\phi \beta _1 S^0}{AB}$, signifies the average rate of infection for susceptible chicken individuals due to one symptomatic chicken individual.

$\beta _2 SC_v$ represents the rate of infection among susceptible chicken individuals per unit time for contaminated vectors. Therefore, the rate of infection for one contaminated vector individual is $\beta _2S$, and the average lifespan of contaminated vectors is $\frac{1}{\mu _v}$. Additionally, contaminated vector individuals originate from susceptible vector individuals infected through contact with symptomatic chicken individuals, i.e., $\beta _3S_vI$. Analogous to the first term, the average rate of infection for susceptible vector individuals due to one symptomatic chicken individual is $\frac{\phi \beta _3 S_v^0}{AB}$. Thus, the second term of $R_0$, $\frac{\phi \beta _2\beta _3 S^0S_v^0 }{\mu _v AB}$, represents the average rate of infection for susceptible chicken individuals due to one contaminated vector individual infected by one symptomatic chicken.

##### Theorem 3.4

*The disease-free equilibrium *$EE=(S^0,E^0,I^0,Q^0,S_v^0,C_v^0)=\left( \frac{\Lambda }{\mu },0,0,0,\frac{\Lambda _v}{\mu _v},0\right)$* is locally asymptotically stable if *$R_0<1$.

##### Proof

The Jacobian matrix of the system (3) at DFE can be computed as below

$$\begin{aligned} J(EE) = \begin{pmatrix} -\mu & 0 & -\beta _1 S^0 & 0 & 0 & -\beta _2S^0\\ 0 & -A & \beta _1 S^0 & 0 & 0 & \beta _2S^0\\ 0 & \phi & -B & 0 & 0 & 0 \\ 0 & 0 & \rho & -\mu & 0 & 0 \\ 0 & 0 & -\beta _3 S_v^0 & 0& -\mu _v & 0\\ 0 & 0 & \beta _3 S_v^0 & 0& 0 & -\mu _v \end{pmatrix}. \end{aligned}$$

By setting $\det (J(EE)-\psi I)=0$, we obtain the eigenvalues:

$$\begin{aligned} \psi _1=\psi _2=-\mu ,\psi _3=-\mu _v, \end{aligned}$$

and the roots of the following characteristic equation:

4 $$\begin{aligned} \psi ^3+a_2\psi ^2 +a_1\psi +a_0=0 \end{aligned}$$

where $$a_2 = A+B+\mu _v > 0$$ , $$a_1 = AB(1-R_0)+\mu _v(A+B)$$$$+\frac{\beta _2\beta _3\phi S^0S_v^0}{\mu _v}$$ , $$a_0 = \mu _vAB(1-R_0) > 0$$ $$\,\text {if}\quad R_0<1$$

By employing the Routh–Hurwitz criteria, we can ensure that all roots of the equation (4) have a negative real component if the following conditions are satisfied:

$$\begin{aligned} a_2>0, a_1>0, a_0>0, a_1a_2>a_0. \end{aligned}$$

It is clear that $a_1>0$ and if $R_0<1$ then $a_2>0, a_0>0$. as well. We need to demonstrate that $a_1a_2>a_0$.

$$\begin{aligned} a_1a_2&=[AB(1-R_0)+\mu _v(A+B)+\frac{\beta _2\beta _3\phi S^0S_v^0}{\mu _v}][A+B+\mu _v]\\&\ge \mu _vAB(1-R_0). \end{aligned}$$

Thus, we establish a robust assurance that all roots of the Eq. (4) possess a negative real component when $R_0 < 1$ Consequently, we conclude that the equilibrium point denoted as *EE* demonstrates local asymptotic stability when $R_0 < 1$, aligning with our intended conclusion. $\square$

#### The global stability of disease-free equilibrium

##### Theorem 3.5

*The disease-free equilibrium *$EE=(S^0,E^0,I^0,Q^0,S_v^0,C_v^0)=\left( \frac{\Lambda }{\mu },0,0,0,\frac{\Lambda _v}{\mu _v},0\right)$* is globally asymptotically stable if *$R_0<1$.

##### Proof

To demonstrate that the disease eventually dies out “global asymptotic stability”, we will utilize a mathematical tool called a Lyapunov function. We will construct a specific function, denoted by *L*, which satisfies certain properties.

Define

$$\begin{aligned} L=\mu _v\phi E+\mu _vAI+\frac{\beta _2\phi \Lambda }{\mu } C_v. \end{aligned}$$

Then,

$$\begin{aligned} \frac{dL}{dt}&=\mu _v\phi \frac{dE}{dt}+\mu _vA\frac{dI}{dt}+\frac{\beta _2\phi \Lambda }{\mu }\frac{dC_v}{dt}\\&=\mu _v\phi \left[ (\beta _1 I+\beta _2C_v)S-AE\right] +\mu _vA\left[ \phi E-BI\right] +\frac{\beta _2\phi \Lambda }{\mu }\left[ \beta _3 S_vI-\mu _vC_v\right] \\&=(\frac{\mu _v\phi \beta _1\Lambda }{\mu } +\frac{\beta _2\beta _3\phi \Lambda \Lambda _v}{\mu \mu _v}-\mu _vAB)I \quad \text {when} \quad S=S^0, S_v=S^0_v\\&=\mu _vAB(R_0-1)I . \end{aligned}$$

So $dL/dt \le 0$, if $R_0 < 1$. Furthermore, $dL/dt= 0$ if $I=0$ or $R_0=1$. From this we see that, $EE=(S^0,E^0,I^0,Q^0,S_v^0,C_v^0)=\left( \frac{\Lambda }{\mu },0,0,0,\frac{\Lambda _v}{\mu _v},0\right)$ is the only singleton in $\{(S^0,E^0,I^0,Q^0,S_v^0,C_v^0)$ $\in \Omega : dL/dt= 0\}$. Therefore by the principle of LaSalle^17^, *EE* is globally asymptotically stable if $R_0<1.$ $\square$

#### Local stability of endemic equilibrium point (EEP)

The EEP is defined as

$$\begin{aligned} EE^*=(S^*,E^*,I^*,Q^*,S_v^*,C_v^*) \end{aligned}$$

where

5 $$\begin{aligned} \begin{aligned} S^*&=\frac{\Lambda }{\beta _1I^*+\beta _2C_v^*+\mu },\\ E^*&=\frac{BI^*}{\phi },\\ Q^*&=\frac{\rho I^*}{\mu },\\ S_v^*&=\frac{\Lambda _v}{\beta _3I^*+\mu _v},\\ C_v^*&=\frac{\beta _3S_v^*I^*}{\mu _v}.\\ \end{aligned} \end{aligned}$$

Since we obtained the steady states as functions of the infected variable $I^*$, we substitute the expression of Eq. (5) into the fifth equation of system (3) to obtain the quadratic equation as follows

6 $$\begin{aligned} b_2I^{*2}+b_1I^*+b_0=0 \end{aligned}$$

where

$$\begin{aligned} b_2&=\mu _v\beta _1\beta _3AB,\\ b_1&=\mu \mu _v\beta _3AB(1-R_0)+\mu _v^2\beta _1AB+\Lambda _v\beta _2\beta _3AB+\frac{\Lambda \Lambda _v\beta _2\beta _3^2\phi }{\mu _v},\\ b_0&=\mu \mu _v^2AB(1-R_0). \end{aligned}$$

As such, the positive endemic equilibrium of nonlinear system (3) are obtained when (6) is solved for the values of $I^*.$ The coefficient $b_2$ is always positive and also, $b_0$ is always positive when $R_0 < 1$ and negative when $R_0 > 1$ . Hence, the following is established.

##### Theorem 3.6

*The model has *

1. *a unique endemic equilibrium if *$R_0 > 1$,
2. *no endemic equilibrium otherwise.*

##### Theorem 3.7

*The endemic equilibrium *$EE^*=(S^*,E^*,I^*,Q^*,S_v^*,C_v^*)$* exists and is locally asymptotically stable if *$R_0>1$.

##### Proof

The Jacobian matrix of the system (3) at $EE^*$ is calculated as

$$\begin{aligned} J(EE^*) = \begin{pmatrix} -\beta _1I^*-\beta _2C_v^*-\mu & 0 & -\beta _1 S^* & 0 & 0 & -\beta _2S^*\\ \beta _1I^*+\beta _2C_v^* & -A & \beta _1 S^* & 0 & 0 & \beta _2S^*\\ 0 & \phi & -B & 0 & 0 & 0 \\ 0 & 0 & \rho & -\mu & 0 & 0 \\ 0 & 0 & -\beta _3 S_v^* & 0& -\beta _3I^*-\mu _v & 0\\ 0 & 0 & \beta _3 S_v^* & 0& \beta _3I^* & -\mu _v \end{pmatrix}. \end{aligned}$$

Setting $\det (J(EE^*)-\xi I)=0$ to obtain the characteristic equation, we get the eigenvalue

$$\begin{aligned} \xi _1=-\mu . \end{aligned}$$

The rest eigenvalues are the roots of the following quintic equation

7 $$\begin{aligned} c_5\xi ^5+c_4\xi ^4+c_3\xi ^3+c_2\xi ^2+c_1\xi +c_0=0, \end{aligned}$$

where

$$\begin{aligned} c_5&=\beta _1\beta _3^2\mu _v^2I^{*3}+(\mu \beta _3^2\mu _v^2+\Lambda _v\beta _2\beta _3^2\mu _v+2\beta _1\beta _3\mu _v^3)I^{*2}+(2\mu \beta _3\mu _v^3+\Lambda _v\beta _2\beta _3\mu _v^2+\beta _1\mu _v^4)I^*+\mu \mu _v^4, \end{aligned}$$

$$\begin{aligned} c_4&=(\beta _1^2\beta _3^2\mu _v^2+\beta _1\beta _3^3\mu _v^2)I^{*4}+(A\beta _1\beta _3^2\mu _v^2+B\beta _1\beta _3^2\mu _v^2+2\mu \beta _1\beta _3^2\mu _v^2+\mu \beta _3^3\mu _v^2+2\Lambda _v\beta _1\beta _2\beta _3^2\mu _v\\&\quad +\Lambda _v\beta _2\beta _3^3\mu _v+2\beta _1^2\beta _3\mu _v^3+4\beta _1\beta _3^2\mu _v^3)I^{*3}+(A\mu \beta _3^2\mu _v^2+A\Lambda _v\beta _2\beta _3^2\mu _v+2A\beta _1\beta _3\mu _v^3+B\mu \beta _3^2\mu _v^2\\&\quad +B\Lambda _v\beta _2\beta _3^2\mu _v+2B\beta _1\beta _3\mu _v^3+\mu ^2\beta _3^2\mu _v^2+2\mu \Lambda _v\beta _2\beta _3^2\mu _v+4\mu \beta _1\beta _3\mu _v^3+4\mu \beta _3^2\mu _v^3+\Lambda _v^2\beta _2^2\beta _3^2\\&\quad +2\Lambda _v\beta _1\beta _2\beta _3\mu _v^2+3\Lambda _v\beta _2\beta _3^2\mu _v^2+\beta _1^2\mu _v^4+5\beta _1\beta _3\mu _v^4)I^{*2}+(2A\mu \beta _3\mu _v^3+A\Lambda _v\beta _2\beta _3\mu _v^2+A\beta _1\mu _v^4\\&\quad +2B\mu \beta _3\mu _v^3+B\Lambda _v\beta _2\beta _3\mu _v^2+B\beta _1\mu _v^4+2\mu ^2\beta _3\mu _v^3+2\mu \Lambda _v\beta _2\beta _3\mu _v^2+2\mu \beta _1\mu _v^4+5\mu \beta _3\mu _v^4\\&\quad +2\Lambda _v\beta _2\beta _3\mu _v^3+2\beta _1\mu _v^5)I^*+A\mu \mu _v^4+B\mu \mu _v^4+\mu ^2\mu _v^4+2\mu \mu _v^5,\end{aligned}$$

$$\begin{aligned} c_3&=\beta _1^2\beta _3^3\mu _v^2I^{*5}+(A\beta _1^2\beta _3^2\mu _v^2+A\beta _1\beta _3^3\mu _v^2+B\beta _1^2\beta _3^2\mu _v^2+B\beta _1\beta _3^3\mu _v^2+2\mu \beta _1\beta _3^3\mu _v^2+2\Lambda _v\beta _1\beta _2\beta _3^3\mu _v\\&\quad +4\beta _1^2\beta _3^2\mu _v^3+\beta _1\beta _3^3\mu _v^3)I^{*4}+(2A\mu \beta _1\beta _3^2\mu _v^2+A\mu \beta _3^3\mu _v^2+2A\Lambda _v\beta _1\beta _2\beta _3^2 \mu _v+A\Lambda _v \beta _2\beta _3^3\mu _v\\&\quad +2A\beta _1^2\beta _3\mu _v^3+4 A\beta _1\beta _3^2\mu _v^3+2B\mu \beta _1\beta _3^2\mu _v^2+B\mu \beta _3^3 \mu _v^2+2 B \Lambda _v \beta _1 \beta _2 \beta _3^2 \mu _v+B \Lambda _v \beta _2 \beta _3^3 \mu _v\\&\quad +2 B\beta _1^2\beta _3\mu _v^3+4 B\beta _1\beta _3^2 \mu _v^3+\mu ^2\beta _3^3\mu _v^2+2\mu \Lambda _v\beta _2\beta _3^3\mu _v+8\mu \beta _1\beta _3^2\mu _v^3+\mu \beta _3^3\mu _v^3+\Lambda _v^2\beta _2^2\beta _3^3\\&\quad +6\Lambda _v\beta _1\beta _2\beta _3^2\mu _v^2+\Lambda _v\beta _2\beta _3^3\mu _v^2+5\beta _1^2\beta _3\mu _v^4+3\beta _1\beta _3^2\mu _v^4)I^{*3}+(A\mu ^2\beta _3^2\mu _v^2+2A\mu \Lambda _v\beta _2\beta _3^2\mu _v\\&\quad +4A\mu *\beta _1\beta _3\mu _v^3+4A\mu \beta _3^2\mu _v^3+A\Lambda _v^2\beta _2^2\beta _3^2+2A\Lambda _v\beta _1\beta _2\beta _3\mu _v^2+3A\Lambda _v\beta _2\beta _3^2\mu _v^2+A\beta _1^2\mu _v^4\\&\quad +5A\beta _1\beta _3\mu _v^4+B\mu ^2\beta _3^2\mu _v^2+2B\mu \Lambda _v\beta _2\beta _3^2\mu _v+4B\mu \beta _1\beta _3\mu _v^3+4B\mu \beta _3^2\mu _v^3+B\Lambda _v^2\beta _2^2\beta _3^2\\&\quad +2B\Lambda _v\beta _1\beta _2\beta _3\mu _v^2+3B\Lambda _v\beta _2\beta _3^2\mu _v^2+B\beta _1^2\mu _v^4+5B\beta _1\beta _3\mu _v^4+4\mu ^2\beta _3^2\mu _v^3+6\mu \Lambda _v\beta _2\beta _3^2 \mu _v^2\\&\quad +10\mu \beta _1\beta _3\mu _v^4+3\mu \beta _3^2 \mu _v^4+2\Lambda _v^2\beta _2^2\beta _3^2\mu _v+4\Lambda _v\beta _1\beta _2\beta _3\mu _v^3+2\Lambda _v\beta _2 \beta _3^2 \mu _v^3+2\beta _1^2\mu _v^5\\&\quad +3\beta _1\beta _3\mu _v^5)I^{*2}+(2A\mu ^2\beta _3\mu _v^3+2A\mu \Lambda _v\beta _2\beta _3\mu _v^2+2A\mu \beta _1\mu _v^4+5A\mu \beta _3\mu _v^4+2A\Lambda _v\beta _2\beta _3\mu _v^3\\&\quad +2A\beta _1\mu _v^5+2B\mu ^2\beta _3\mu _v^3+2B\mu \Lambda _v\beta _2\beta _3\mu _v^2+2B\mu \beta _1\mu _v^4+5B\mu \beta _3\mu _v^4+2B\Lambda _v\beta _2\beta _3\mu _v^3+2B\beta _1\mu _v^5\\&\quad +5\mu ^2\beta _3\mu _v^4+4\mu \Lambda _v\beta _2\beta _3\mu _v^3+4\mu \beta _1\mu _v^5+3\mu \beta _3\mu _v^5+\Lambda \Lambda _v\beta _2\beta _3^2\phi \mu _v+\Lambda _v\beta _2\beta _3\mu _v^4+\beta _1\mu _v^6)I^*\\&\quad +A\mu ^2\mu _v^4+2A\mu \mu _v^5+B\mu ^2\mu _v^4+2B\mu \mu _v^5+2\mu ^2\mu _v^5+\mu \mu _v^6+\Lambda \Lambda _v\beta _2\beta _3\phi \mu _v^2, \end{aligned}$$

$$\begin{aligned} c_2&=(A\beta _1^2\beta _3^3\mu _v^2+B\beta _1^2\beta _3^3\mu _v^2+\beta _1^2\beta _3^3\mu _v^3)I^{*5}+(AB\beta _1^2\beta _3^2\mu _v^2+2A\mu \beta _1\beta _3^3\mu _v^2+2A\Lambda _v\beta _1\beta _2\beta _3^3\mu _v\\&\quad +4A\beta _1^2\beta _3^2\mu _v^3+A\beta _1\beta _3^3\mu _v^3+2B\mu \beta _1\beta _3^3\mu _v^2+2B\Lambda _v\beta _1\beta _2\beta _3^3\mu _v+4B\beta _1^2\beta _3^2\mu _v^3+B\beta _1\beta _3^3\mu _v^3+2\mu \beta _1\beta _3^3\mu _v^3\\&\quad +2\Lambda _v\beta _1\beta _2\beta _3^3\mu _v^2+3\beta _1^2\beta _3^2\mu _v^4)I^{*4}+(AB\mu \beta _1\beta _3^2\mu _v^2+2AB\Lambda _v\beta _1\beta _2\beta _3^2\mu _v+2AB\beta _1^2\beta _3\mu _v^3+A\mu ^2\beta _3^3\mu _v^2\\&\quad +2A\mu \Lambda _v\beta _2\beta _3^3\mu _v+8A\mu \beta _1\beta _3^2\mu _v^3+A\mu \beta _3^3\mu _v^3+A\Lambda _v^2\beta _2^2\beta _3^3+6A\Lambda _v\beta _1\beta _2\beta _3^2\mu _v^2+A\Lambda _v\beta _2\beta _3^3\mu _v^2\\&\quad +5A\beta _1^2\beta _3\mu _v^4+3A\beta _1\beta _3^2\mu _v^4+B\mu ^2\beta _3^3\mu _v^2+2B\mu \Lambda _v\beta _2\beta _3^3\mu _v+8B\mu \beta _1\beta _3^2\mu _v^3+B\mu \beta _3^3\mu _v^3+B\Lambda _v^2\beta _2^2\beta _3^3\\&\quad +6B\Lambda _v\beta _1\beta _2\beta _3^2\mu _v^2+B\Lambda _v\beta _2\beta _3^3\mu _v^2+5B\beta _1^2\beta _3\mu _v^4+3B\beta _1\beta _3^2\mu _v^4+\mu ^2\beta _3^3\mu _v^3+2\mu \Lambda _v\beta _2\beta _3^3\mu _v^2+6\mu \beta _1\beta _3^2\mu _v^4\\&\quad +\Lambda _v^2\beta _2^2\beta _3^3\mu _v+4\Lambda _v\beta _1\beta _2\beta _3^2\mu _v^3+3\beta _1^2\beta _3\mu _v^5)I^{*3}+(AB\mu \Lambda _v\beta _2\beta _3^2\mu _v+2AB\mu \beta _1\beta _3\mu _v^3+AB\Lambda _v^2\beta _2^2\beta _3^2\\&\quad +2AB\Lambda _v\beta _1\beta _2\beta _3\mu _v^2+AB\beta _1^2\mu _v^4+4A\mu ^2\beta _3^2\mu _v^3+6A\mu \Lambda _v\beta _2\beta _3^2\mu _v^2+10A\mu \beta _1\beta _3\mu _v^4+3A\mu \beta _3^2\mu _v^4\\&\quad +2A\Lambda _v^2\beta _2^2\beta _3^2\mu _v+4A\Lambda _v\beta _1\beta _2\beta _3\mu _v^3+2A\Lambda _v\beta _2\beta _3^2\mu _v^3+2A\beta _1^2\mu _v^5+3A\beta _1\beta _3\mu _v^5+4B\mu ^2\beta _3^2\mu _v^3\\&\quad +6B\mu \Lambda _v\beta _2\beta _3^2\mu _v^2+10B\mu \beta _1\beta _3\mu _v^4+3B\mu \beta _3^2\mu _v^4+2B\Lambda _v^2\beta _2^2\beta _3^2\mu _v+4B\Lambda _v\beta _1\beta _2\beta _3\mu _v^3+2B\Lambda _v\beta _2\beta _3^2\mu _v^3\\&\quad +2B\beta _1^2\mu _v^5+3B\beta _1\beta _3\mu _v^5+3\mu ^2\beta _3^2\mu _v^4+4\mu \Lambda _v\beta _2\beta _3^2\mu _v^3+6\mu \beta _1\beta _3\mu _v^5+\Lambda _v^2\beta _2^2\beta _3^2\mu _v^2+2\Lambda _v\beta _1\beta _2\beta _3\mu _v^4\\&\quad +\beta _1^2\mu _v^6+\Lambda \Lambda _v\beta _2\beta _3^3\phi \mu _v^3)I^{*2}+(AB\mu \Lambda _v\beta _2\beta _3\mu _v^2+AB\mu \beta _1\mu _v^4+5A\mu ^2\beta _3\mu _v^4+4A\mu \Lambda _v\beta _2\beta _3\mu _v^3\\&\quad +4A\mu \beta _1\mu _v^5+3A\mu \beta _3\mu _v^5+A\Lambda _v\beta _2\beta _3\mu _v^4+A\beta _1\mu _v^6+5B\mu ^2\beta _3\mu _v^4+4B\mu \Lambda _v\beta _2\beta _3\mu _v^3+4B\mu \beta _1\mu _v^5\\&\quad +3B\mu \beta _3\mu _v^5+B\Lambda _v\beta _2\beta _3\mu _v^4+B\beta _1\mu _v^6+3\mu ^2\beta _3\mu _v^5+2\mu \Lambda _v\beta _2\beta _3\mu _v^4+2\mu \beta _1\mu _v^6+\mu \Lambda \Lambda _v\beta _2\beta _3^2\phi \mu _v\\ &\quad +2\Lambda \Lambda _v\beta _2\beta _3^2\phi \mu _v^2)I^*+2A\mu ^2\mu _v^5+A\mu \mu _v^6+2B\mu ^2\mu _v^5+B\mu \mu _v^6+\mu ^2\mu _v^6+\mu \Lambda \Lambda _v\beta _2\beta _3\phi \mu _v^2\\&\quad +\Lambda \Lambda _v\beta _2\beta _3\phi \mu _v^3, \end{aligned}$$

$$\begin{aligned}c_1&=(AB\beta _1^2\beta _3^3\mu _v^2+A\beta _1^2\beta _3^3\mu _v^3+B\beta _1^2\beta _3^3\mu _v^3)I^{*5}+(AB\mu \beta _1\beta _3^3\mu _v^2+2AB\Lambda _v\beta _1\beta _2\beta _3^3\mu _v+4AB\beta _1^2\beta _3^2\mu _v^3\\&\quad +2A\mu \beta _1\beta _3^3\mu _v^3+2A\Lambda _v\beta _1\beta _2\beta _3^3\mu _v^2+3A\beta _1^2\beta _3^2\mu _v^4+2B\mu \beta _1\beta _3^3\mu _v^3+2B\Lambda _v\beta _1\beta _2\beta _3^3\mu _v^2+3B\beta _1^2\beta _3^2\mu _v^4)I^{*4}\\&\quad +(AB\mu \Lambda _v\beta _2\beta _3^3\mu _v+4AB\mu \beta _1\beta _3^2\mu _v^3+AB\Lambda _v^2\beta _2^2\beta _3^3+6AB\Lambda _v\beta _1\beta _2\beta _3^2\mu _v^2+5AB\beta _1^2\beta _3\mu _v^4+A\mu ^2\beta _3^3\mu _v^3\\&\quad +2A\mu \Lambda _v\beta _2\beta _3^3\mu _v^2+6A\mu \beta _1\beta _3^2\mu _v^4+A\Lambda _v^2\beta _2^2\beta _3^3\mu _v+4A\Lambda _v\beta _1\beta _2\beta _3^2\mu _v^3+3A\beta _1^2\beta _3\mu _v^5+B\mu ^2\beta _3^3\mu _v^3\\&\quad +2B\mu \Lambda _v\beta _2\beta _3^3\mu _v^2+6B\mu \beta _1\beta _3^2\mu _v^4+B\Lambda _v^2\beta _2^2\beta _3^3\mu _v+4B\Lambda _v\beta _1\beta _2\beta _3^2\mu _v^3+3B\beta _1^2\beta _3\mu _v^5)I^{*3}\\&\quad +(3AB\mu \Lambda _v\beta _2\beta _3^2\mu _v^2+5AB\mu \beta _1\beta _3\mu _v^4+2AB\Lambda _v^2\beta _2^2\beta _3^2\mu _v+4AB\Lambda _v\beta _1\beta _2\beta _3\mu _v^3+2AB\beta _1^2\mu _v^5\\&\quad +3A\mu ^2\beta _3^2\mu _v^4+4A\mu \Lambda _v\beta _2\beta _3^2\mu _v^3+6A\mu \beta _1\beta _3\mu _v^5+A\Lambda _v^2\beta _2^2\beta _3^2\mu _v^2+2A\Lambda _v\beta _1\beta _2\beta _3\mu _v^4+A\beta _1^2\mu _v^6\\&\quad +3B\mu ^2\beta _3^2\mu _v^4+4B\mu \Lambda _v\beta _2\beta _3^2\mu _v^3+6B\mu \beta _1\beta _3\mu _v^5+B\Lambda _v^2\beta _2^2\beta _3^2\mu _v^2+2B\Lambda _v\beta _1\beta _2\beta _3\mu _v^4+B\beta _1^2\mu _v^6\\&\quad +\mu \Lambda \Lambda _v\beta _2\beta _3^3\phi \mu _v+\Lambda \Lambda _v\beta _2\beta _3^3\phi \mu _v^2)I^{*2}+(2AB\mu \Lambda _v\beta _2\beta _3\mu _v^3+2AB\mu \beta _1\mu _v^5+3A\mu ^2\beta _3\mu _v^5\\&\quad +2A\mu \Lambda _v\beta _2\beta _3\mu _v^4+2A\mu \beta _1\mu _v^6+3B\mu ^2\beta _3\mu _v^5+2B\mu \Lambda _v\beta _2\beta _3\mu _v^4+2B\mu \beta _1\mu _v^6+2\mu \Lambda \Lambda _v\beta _2\beta _3^2\phi \mu _v^2\\&\quad +\Lambda \Lambda _v\beta _2\beta _3^2\phi \mu _v^3)I^*+A\mu ^2\mu _v^6+B\mu ^2\mu _v^6+\mu \Lambda \Lambda _v\beta _2\beta _3\phi \mu _v^3,\end{aligned}$$

$$\begin{aligned} c_0&=AB\beta _1^2\beta _3^3\mu _v^3I^{*5}+(AB\mu \beta _1\beta _3^3\mu _v^3+2AB\Lambda _v\beta _1\beta _2\beta _3^3\mu _v^2+3AB\beta _1^2\beta _3^2\mu _v^4)I^{*4}+(AB\mu \Lambda _v\beta _2\beta _3^3\mu _v^2\\&\quad +3AB\mu \beta _1\beta _3^2\mu _v^4+AB\Lambda _v^2\beta _2^2\beta _3^3\mu _v+4AB\Lambda _v\beta _1\beta _2\beta _3^2\mu _v^3+3AB\beta _1^2\beta _3\mu _v^5)I^{*3}+(2AB\mu \Lambda _v\beta _2\beta _3^2\mu _v^3\\&\quad +4AB\mu \beta _1\beta _3\mu _v^5+AB\Lambda _v^2\beta _2^2\beta _3^2\mu _v^2+2AB\Lambda _v\beta _1\beta _2\beta _3\mu _v^4+AB\beta _1^2\mu _v^6+\mu \Lambda \Lambda _v\beta _2\beta _3^3\phi \mu _v^2)I^{*2}\\&\quad +(AB\mu \Lambda _v\beta _2\beta _3\mu _v^4+AB\mu \beta _1\mu _v^6+\mu \Lambda \Lambda _v\beta _2\beta _3^2\phi \mu _v)I^*. \end{aligned}$$

Note that, $c_i > 0$ for $i = 0,1,2,...,5$ if $I^*> 0$ this mean that $R_0 > 1$. By the Routh–Hurwitz criteria, we can confirm that all roots of the above quintic equation have a negative real part when satisfy the following conditions.

$$\begin{aligned} & c_5>0, c_4>0, c_3>0, c_2>0, c_1>0, c_0>0, c_3c_4>c_2c_5, c_2c_3c_4>c_1c_4^2+c_2^2c_5, \\ & c_1c_2c_3c_4+2c_0c_1c_4c_5+c_0c_2c_3c_5 >c_1^2c_4^2+c_0c_3^2c_4+c_1c_3^2c_5+c_0^2c_5^2. \end{aligned}$$

As a result, $R_0>1$. Therefore, $EE^*$ is locally asymptotically stable whenever it exists, as intended conclusion. $\square$

#### The global stability of endemic equilibrium

##### Theorem 3.8

*The endemic equilibrium point *$EE^*=(S^*,E^*,I^*,Q^*,S_v^*,C_v^*)$* exists and is globally asymptotically stable if *$R_0>1$* and *$m<n$.

##### Proof

In order to analyze the long-term behavior “global asymptotic stability” of the state where the disease persists, we will employ a mathematical tool called a Lyapunov function. We will define a specific function, denoted by *L*, as follows:

$$\begin{aligned} L(S^*,E^*,I^*,Q^*,S_v^*,C_v^*) =&\left( S-S^*\ln \frac{S}{S^*}\right) +\left( E-E^*\ln \frac{E}{E^*}\right) +\left( I-I^*\ln \frac{I}{I^*}\right) \\&+\left( Q-Q^*\ln \frac{Q}{Q^*}\right) +\left( S_v-S_v^*\ln \frac{S_v}{S_v^*}\right) +\left( C_v-C_v^*\ln \frac{C_v}{C_v^*}\right) . \end{aligned}$$

Note that $EE^*$ exists if $R_0>1$. By direct calculating the derivative of *L* along the system (3) we have,

$$\begin{aligned} \frac{dL}{dt}&=\left( 1-\frac{S^*}{S}\right) \frac{dS}{dt}+\left( 1-\frac{E^*}{E}\right) \frac{dE}{dt}+\left( 1-\frac{I^*}{I}\right) \frac{dI}{dt}+\left( 1-\frac{Q^*}{Q}\right) \frac{dQ}{dt}+\left( 1-\frac{S_v^*}{S_v}\right) \frac{dS_v}{dt}+\left( 1-\frac{C_v^*}{C_v}\right) \frac{dC_v}{dt}\\&=\left( 1-\frac{S^*}{S}\right) [\Lambda -(\beta _1 I+\beta _2C_v)S -\mu S]+\left( 1-\frac{E^*}{E}\right) [(\beta _1 I+\beta _2C_v)S-AE]\\&\quad +\left( 1-\frac{I^*}{I}\right) [\phi E-BI] +\left( 1-\frac{Q^*}{Q}\right) [\rho I-\mu Q]+\left( 1-\frac{S_v^*}{S_v}\right) [\Lambda _v-\beta _3 S_vI -\mu _v S_v]\\&\quad +\left( 1-\frac{C_v^*}{C_v}\right) [\beta _3 S_vI-\mu _vC_v]\\&=[\Lambda +\Lambda _v+\mu S^*+AE^*+BI^*+\mu Q^*+\mu _vS_v^*+\mu _vC_v^*+\beta _1IS^*+\beta _2C_vS^*+\beta _3IS_v^*\\&\quad +\phi E+\rho I] -\big [\frac{\Lambda S^*}{S} +\frac{\beta _1 SIE^*}{E}+\frac{\beta _2 SC_vE^*}{E}+\frac{\phi EI^*}{I}+\frac{\rho IQ^*}{Q}+\frac{\Lambda _vS_v^*}{S_v}+\frac{\beta _3S_vIC_v^* }{C_v}\\&\quad +\mu _vS_v+\mu S+AE+BI+\mu Q+\mu _vC_v\big ]. \end{aligned}$$

Thus, by combining positive and negative terms, we get

$$\begin{aligned} \frac{dL}{dt}=m-n. \end{aligned}$$

Here,

$$\begin{aligned} m&\equiv \Lambda +\Lambda _v+\mu S^*+AE^*+BI^*+\mu Q^*+\mu _vS_v^*+\mu _vC_v^*+\beta _1IS^*+\beta _2C_vS^*+\beta _3IS_v^*\\&\quad +\phi E+\rho I,\\ n&\equiv \frac{\Lambda S^*}{S} +\frac{\beta _1 SIE^*}{E}+\frac{\beta _2 SC_vE^*}{E}+\frac{\phi EI^*}{I}+\frac{\rho IQ^*}{Q}+\frac{\Lambda _vS_v^*}{S_v}+\frac{\beta _3S_vIC_v^* }{C_v}+\mu _vS_v+\mu S+AE\\&\quad +BI+\mu Q+\mu _vC_v. \end{aligned}$$

Thus, if $m<n,$ then *dL*/*dt* is non-positive. This change is zero only when all the system variables are at their specific equilibrium point $(S=S^*, E=E^*, I=I^*, Q=Q^*, S_v=S_v^*, C_v=C_v^*)$. As a result, the greatest compact invariant set in $\{(S^*,E^*,I^*,Q^*,S_v^*,C_v^*) \in \Omega : dL/dt= 0\}$ is the singleton $EE^*$, which represents the endemic equilibrium of the system (3). By the LaSalle’s invariant principle^17^, we have that the endemic equilibrium $EE^*$ exists and is globally asymptotically stable in $\Omega$ if $R_0>1$ and $m<n$. $\square$

## SEIHR model with impulsive vaccination

In this section, the impulsive mathematical model is introduced. The vaccination will apply to population in periodic style. The main propose of this section is the conditions that cause the disease die out when the population get vaccine.

### Preliminaries

Let

$$\begin{aligned} G:{\mathbb {R}}_+\times {\mathbb {R}}_+^7\rightarrow {\mathbb {R}}_+, \end{aligned}$$

where $${\mathbb {R}}_+=[0,\infty ), {\mathbb {R}}_+^7$$$$=\{X\in {\mathbb {R}}^7:X=(S,V,E,I,Q,S_v,C_v),$$ $$S\ge 0, V\ge 0,\,$$$$E\ge 0, I\ge 0,\,$$$$Q\ge 0,$$ $$S_v\ge 0,C_v\ge 0\}$$. The map defined by the right hand side of system (1) is denoted by $$F=(F_1,F_2,...,F_7)$$.

#### Definition 4.1

(^18^) The function *G* is said to belong to class $G_0$ if *G* is continuous in $(nT,(n+1)T]\times {\mathbb {R}}_+^7\rightarrow {\mathbb {R}}_+$ and for each $X\in {\mathbb {R}}_+^7,n\in {\mathbb {Z}}_+,$

$$\begin{aligned} \lim _{(t,Y)\rightarrow (nT^+,X)} G(t,Y)=G(nT^+,X) \end{aligned}$$

exits and locally Lipschitzian in *X*.

Suppose $G \in G_0.$ For $t \in (nT,(n+1)T]\times {\mathbb {R}}_+^7,$ the upper right derivative of *G*(*t*, *X*) with respect to system (1) and (2) is defined by

$$\begin{aligned} {D^{+}}G(t,X)=\limsup _{h \rightarrow 0^+}\dfrac{1}{h}[G(t+h,X+hF(t,X))-G(t,X)]. \end{aligned}$$

The solution of system (1) and (2), $X(t)=(S,V,E,I,Q,S_v,C_v)$, is assumed to be a piecewise continuous function. It means that, $X(t): {\mathbb {R}}_+ \rightarrow {\mathbb {R}}_+^7, X(t)$ is continuous on $(nT,(n+1)T]$, $n\in {\mathbb {Z}}_+$ and $\lim _{t\rightarrow nT^+}X(t)=X(nT^+)$ exists. Therefore, the smoothness properties of *F* ensure the existence and uniqueness of solution to (1)–(2)^19^.

#### Lemma 4.2

*Suppose *$X(t)=(S(t),V(t),E(t),I(t),Q(t),S_v(t),C_v(t))$* is a solution of the system *(1)* and *(2)* with the initial value *$X({{0}^{+}})\ge 0$*. Then the solution *$X(t)\ge 0$* for all *$t\ge 0$.

#### Proof

For $t\ne nT$: $dS/dt>0$ whenever $S(t)=0$. It means that *S*(*t*) is non-negative solutions.

For $t= nT$: $S(nT^+)=(1-\omega )S(nT)$. We can conclude that *S*(*t*) is non-negative solutions since $S(nT)\ge 0$ (by Case $t\ne nT$) and $0<\omega <1$.

The similar approach can be used to show $V(t), E(t), I(t), Q(t), S_v(t)$ and $C_v(t)$. The proof is completed. $\square$

### The epidemic model under periodic impulsive vaccination

If we assume the disease has completely stopped spreading this means that the rate of change for each population in the system (1) is zero, and there are currently no exposed chickens, infected chickens, or contaminated vectors, while the quarantined compartments are also empty, then the system reaches the disease-free steady-state. This state is described as below:

$$\begin{aligned} S_{0*}^+&=\lim _{n\rightarrow \infty } S(nT^+)=\dfrac{\Lambda (1-\omega )(1-e^{-(\varepsilon +\mu )T})}{\mu [1-(1-\omega )e^{-(\varepsilon +\mu )T}]},\\ S_{0*}&=\lim _{n\rightarrow \infty } S(nT)=\frac{S_{0*}^+}{1-\omega }=\dfrac{\Lambda (1-e^{-(\varepsilon +\mu )T})}{\mu [1-(1-\omega )e^{-(\varepsilon +\mu )T}]},\\ V_{0*}^+&=N_{0*}-S_{0*}^+=\dfrac{\Lambda \omega }{\mu [1-(1-\omega )e^{-(\varepsilon +\mu )T}]},\\ V_{0*}&=N_{0*}-S_{0*}=\dfrac{\Lambda \omega e^{-(\varepsilon +\mu )T})}{\mu [1-(1-\omega )e^{-(\varepsilon +\mu )T}]}, \end{aligned}$$

with $$E_{0*}^+=E_{0*}=I_{0*}^+=I_{0*}=Q_{0*}^+=Q_{0*}=C_{v0*}^+=C_{v0*}=0$$ and $$S_{v0*}^+=S_{v0*}=\frac{\Lambda _v}{\mu _v}.$$

To analyze the dynamics of the impulsive vaccination, we first analyze the following the susceptible-vaccine subsystem at $(E=I=Q=C_v=0)$.

For $$t \ne nT,$$

8 $$\begin{aligned} \frac{dS}{dt}= & \Lambda -\mu S+\varepsilon V, \end{aligned}$$

9 $$\begin{aligned} \frac{dV}{dt}= & -(\varepsilon +\mu )V, \end{aligned}$$

10 $$\begin{aligned} \frac{dS_v}{dt}= & \Lambda _v -\mu _v S_v. \end{aligned}$$

For $$t = nT,$$

11 $$\begin{aligned} S(nT^+)= & (1-\omega )S(nT), \end{aligned}$$

12 $$\begin{aligned} V(nT^+)= & V(nT)+\omega S(nT), \end{aligned}$$

13 $$\begin{aligned} S_v(nT^+)= & S_v(nT), \end{aligned}$$

14 $$\begin{aligned} S(0^+)= & S_0, \end{aligned}$$

15 $$\begin{aligned} V(0^+)= & V_0, \end{aligned}$$

16 $$\begin{aligned} S_v(0^+)= & S_{v0}. \end{aligned}$$

The system (8)–(13) has a periodic solution

$$\begin{aligned} \tilde{S}(t)=\dfrac{\Lambda }{\mu }\left( 1-\dfrac{\omega e^{-(\varepsilon +\mu )(t-nT)}}{1-(1-\omega )e^{-(\varepsilon +\mu )T}}\right) , \tilde{V}(t) = \dfrac{\Lambda \omega e^{-(\varepsilon +\mu )(t-nT)}}{\mu \left( 1-(1-\omega )e^{-(\varepsilon +\mu )T}\right) }, \tilde{S}_v(t)=\frac{\Lambda _v}{\mu _v}, \end{aligned}$$

with $$\tilde{S}(0^+)=\dfrac{\Lambda }{\mu }\left( 1-\dfrac{\omega }{1-(1-\omega )e^{-(\varepsilon +\mu )T}}\right) >0,$$ $$\tilde{V}(0^+) = \dfrac{\Lambda \omega }{\mu \left( 1-(1-\omega )e^{-(\varepsilon +\mu )T}\right) }>0$$ for $$t \in (nT,(n+1)T], \forall n \in {\mathbb {Z}}_+.$$

Therefore, the positive solution of (8)–(16) is

$$\begin{aligned} & S(t)=\left( S_0-\dfrac{\Lambda }{\mu }\left( 1-\dfrac{\omega }{1-(1-\omega )e^{-(\varepsilon +\mu )T}}\right) \right) e^{-(\varepsilon +\mu )t}+\tilde{S}(t), t \in (nT,(n+1)T],\\ & V(t)=\left( V_0-\dfrac{\Lambda \omega }{\mu \left( 1-(1-\omega )e^{-(\varepsilon +\mu )T}\right) }\right) e^{-(\varepsilon +\mu )t}+\tilde{V}(t), t \in (nT,(n+1)T].\\ & S_v(t)=\tilde{S}_v(t), t \in (nT,(n+1)T. \end{aligned}$$

#### Lemma 4.3

*The system *(8)–(16)* has a positive periodic solution *$(\tilde{S}(t),\tilde{V}(t),\tilde{S}_v(t)),$* and *$(S(t),V(t),S_v(t))$ $\rightarrow$ $(\tilde{S}(t),\tilde{V}(t),\tilde{S}_v(t))$* as *$t \rightarrow \infty$* for every solution *$(S(t),V(t),S_v(t)).$

Therefore,

$$(\tilde{S}(t),\tilde{V}(t),0,0,0,\tilde{S}_v(t),0)$$

$=\left( \dfrac{\Lambda }{\mu }\left( 1-\dfrac{\omega e^{-(\varepsilon +\mu )(t-nT)}}{1-(1-\omega )e^{-(\varepsilon +\mu )T}}\right) ,\dfrac{\Lambda \omega e^{-(\varepsilon +\mu )(t-nT)}}{\mu \left( 1-(1-\omega )e^{-(\varepsilon +\mu )T}\right) },0,0, 0,\dfrac{\Lambda _v}{\mu _v},0\right)$ is a periodic solution of the system (1) and (2) at the absence of *E*, *I*, *Q*,  and $C_v$ for $t \in (nT,(n+1)T], n \in {\mathbb {Z}}_+$ with

$$\begin{aligned} & \tilde{S}(nT^+)=\tilde{S}(0^+)=\dfrac{\Lambda }{\mu }\left( 1-\dfrac{\omega }{1-(1-\omega )e^{-(\varepsilon +\mu )T}}\right) ,\\ & \tilde{V}(nT^+)=\tilde{V}(0^+)=\dfrac{\Lambda \omega }{\mu \left( 1-(1-\omega )e^{-(\varepsilon +\mu )T}\right) } \end{aligned}$$

and

$$\begin{aligned} \tilde{S_v}(nT^+)=\tilde{S_v}(0^+)=\frac{\Lambda _v}{\mu _v}. \end{aligned}$$

#### Theorem 4.4

*The disease-free periodic solution* $(\tilde{S}(t),\tilde{V}(t),0,0,0,\tilde{S}_v(t),0)$ *is locally asymptotically stable if the following condition holds:*

17 $$\begin{aligned} AB\mu _vT>\int _{0}^{T} \tilde{S}\phi \tilde{S}_v\beta _2\beta _3+\tilde{S}\phi \beta _1\mu _v \,dt. \end{aligned}$$

#### Proof

Let us consider a small perturbation

$$\begin{aligned} \begin{aligned} S(t)&= \tilde{S}(t)+k_1(t), \\ V(t)&= \tilde{V}(t)+k_2(t), \\ E(t)&=k_3(t), \\ I(t)&= k_4(t), \\ Q(t)&= k_5(t), \\ S_v(t)&=\tilde{S}_v(t)+k_6(t),\\ C_v(t)&=k_7(t),\\ \end{aligned} \end{aligned}$$

from the point $(\tilde{S}(t),\tilde{V}(t),0,0,0,\tilde{S}_v(t),0).$ Then

$$\begin{aligned} \begin{pmatrix} k_1(t) \\ k_2(t)\\ k_3(t)\\ k_4(t)\\ k_5(t)\\ k_6(t)\\ k_7(t) \end{pmatrix} =\Phi (t) \begin{pmatrix} k_1(0) \\ k_2(0)\\ k_3(0)\\ k_4(0)\\ k_5(0)\\ k_6(0) \\ k_7(0) \end{pmatrix},\ \ 0<t<T \end{aligned}$$

where $$\Phi (t)$$ satisfies

$$\begin{aligned} \dfrac{d\Phi (t)}{dt} = \begin{pmatrix} -\mu & \varepsilon & 0& -\beta _1\tilde{S} & 0 & 0& -\beta _2\tilde{S}\\ 0 & -(\varepsilon +\mu )& 0& 0& 0& 0& 0\\ 0& 0& -A & \beta _1\tilde{S} & 0 & 0& \beta _2\tilde{S}\\ 0 & 0& \phi & -B & 0 & 0& 0\\ 0 & 0& 0 & \rho & -\mu & 0& 0\\ 0 & 0& 0 & -\beta _3\tilde{S}_v & 0 & -\mu _v& 0\\ 0 & 0& 0 & \beta _3\tilde{S}_v & 0 & 0& -\mu _v \end{pmatrix}\Phi (t). \ \end{aligned}$$

Since all columns of matrix $\Phi (t)$ are particularly linearly independent solutions to the initial conditions $\Phi (0)=I_7$. The fundamental matrix $\Phi (t)=\Phi (t+T)$ of the seven-order differential system is nonsingular for all time that is defined as the monodromy matrix $\Phi (T)$ for any $t=nT$. We can write $\Phi (T)$ by the following form:

$$\begin{aligned} \Phi (T)=Diag\left( e^{-\mu T}, e^{-(\varepsilon +\mu ) T},e^{-\mu _vT}, e^{-\mu T},e^{\int _{0}^{T} J_1(t) \,dt}, e^{\int _{0}^{T} J_2(t) \,dt}, e^{\int _{0}^{T} J_3(t) \,dt}\right) \end{aligned}$$

where $J_1(t), J_2(t),$ and $J_3(t)$ are the roots of

$$\begin{aligned} J^3+q_2J^2+q_1J+q_0=0 \end{aligned}$$

where

$$\begin{aligned} q_2&\equiv A+B+\mu _v\\ q_1&\equiv AB+A\mu _v+B\mu _v-\tilde{S}\phi \beta _1\\ q_0&\equiv AB\mu _v -\tilde{S}\phi \tilde{S}_v\beta _2\beta _3-\tilde{S}\phi \beta _1\mu _v. \end{aligned}$$

Linearization of (2) yields

$$\begin{aligned} \begin{pmatrix} k_1(nT^+) \\ k_2(nT^+)\\ k_3(nT^+)\\ k_4(nT^+)\\ k_5(nT^+)\\ k_6(nT^+)\\ k_7(nT^+) \end{pmatrix} = \begin{pmatrix} 1-\omega & 0& 0& 0& 0& 0& 0\\ \omega & 1& 0& 0& 0& 0& 0\\ 0& 0& 1 & 0& 0& 0& 0\\ 0& 0& 0 & 1& 0& 0& 0\\ 0& 0& 0 & 0& 1& 0& 0\\ 0& 0& 0 & 0& 0& 1& 0\\ 0& 0& 0 & 0& 0& 0& 1\\ \end{pmatrix}\begin{pmatrix} k_1(nT) \\ k_2(nT)\\ k_3(nT)\\ k_4(nT)\\ k_5(nT)\\ k_6(nT)\\ k_7(nT) \end{pmatrix}. \end{aligned}$$

By applying the Floquet theory, the solution $(\tilde{S}(t),\tilde{V}(t),0,0,0,\tilde{S}_v(t),0)$ is locally stable if the modulus of all eigenvalues of matrix *P* is less than 1 when matrix *P* can be written by

$$\begin{aligned} P = \begin{pmatrix} 1-\omega & 0& 0& 0& 0& 0& 0\\ \omega & 1& 0& 0& 0& 0& 0\\ 0& 0& 1 & 0& 0& 0& 0\\ 0& 0& 0 & 1& 0& 0& 0\\ 0& 0& 0 & 0& 1& 0& 0\\ 0& 0& 0 & 0& 0& 1& 0\\ 0& 0& 0 & 0& 0& 0& 1 \end{pmatrix}\Phi (T). \end{aligned}$$

Note that the eigenvalues of *P* are

$$\begin{aligned} \begin{aligned} \eta _1&= (1-\omega )\text {exp}(-\mu T), \\ \eta _2&= \text {exp}(-(\varepsilon +\mu ) T), \\ \eta _3&= \text {exp}(-\mu _vT), \\ \eta _4&= \text {exp}(-\mu T), \\ \eta _5&= \text {exp}\left( \int _{0}^{T} J_1(t) \,dt\right) , \\ \eta _6&= \text {exp}\left( \int _{0}^{T} J_2(t) \,dt\right) ,\\ \eta _7&= \text {exp}\left( \int _{0}^{T} J_3(t) \,dt\right) . \end{aligned} \end{aligned}$$

Since (17) hold, then the modulus of all eigenvalues is less than 1. According to this, for sufficiently tiny beginning conditions, the periodic solution is locally asymptotically stable. $\square$

## Numerical simulations

We proceed to express the SC-SEIQ and SC-SVEIQ models through numerical simulations. The solutions of the proposed model system has been carried out by using MATLAB, leveraging packages such as ode45 for solving ordinary differential equations and ode15s for addressing impulsive differential equations. For the example, we utilize the parameter values of the Newcastle disease outlined in Table 1 for our simulations.

**Table 1:** Parameter values.

Parameter	Value	Source	Unit
*S*(0)	20,000	^20^	Individuals
*V*(0)	0	^20^	Individuals
*E*(0)	120	^20^	Individuals
*I*(0)	500	^20^	Individuals
*Q*(0)	0	Assumed	Individuals
$S_v(0)$	300,000	^20^	Individuals
$C_v(0)$	400	^20^	Individuals
$\Lambda$	2.062	Calculated^20^	Individuals$\times$Day$^{-1}$
$\Lambda _v$	30.04	Calculated^20^	Individuals$\times$Day$^{-1}$
$\beta _1$	$4.06\times 10^{-6}-1.45\times 10^{-4}$	^21^	Individuals$^{-1}\times$Day$^{-1}$
$\beta _2$	7$\times 10^{-7}$	Calculated^22,23^	Individuals$^{-1}\times$Day$^{-1}$
$\beta _3$	7$\times 10^{-7}$	Assumed	Individuals$^{-1}\times$Day$^{-1}$
$\mu$	5.8$\times 10^{-4}-0.00136$	^24,25^	Day$^{-1}$
$\mu _v$	5.8$\times 10^{-4}-0.00136$	^24,25^	Day$^{-1}$
$\varepsilon$	0.02	^26^	Day$^{-1}$
$\phi$	0.067–0.625	^27,28^	Day$^{-1}$
$\rho$	0–1	Assumed	Day$^{-1}$
$\delta$	1.989$\times 10^{-2}$	^29^	Day$^{-1}$

**Fig. 2:**
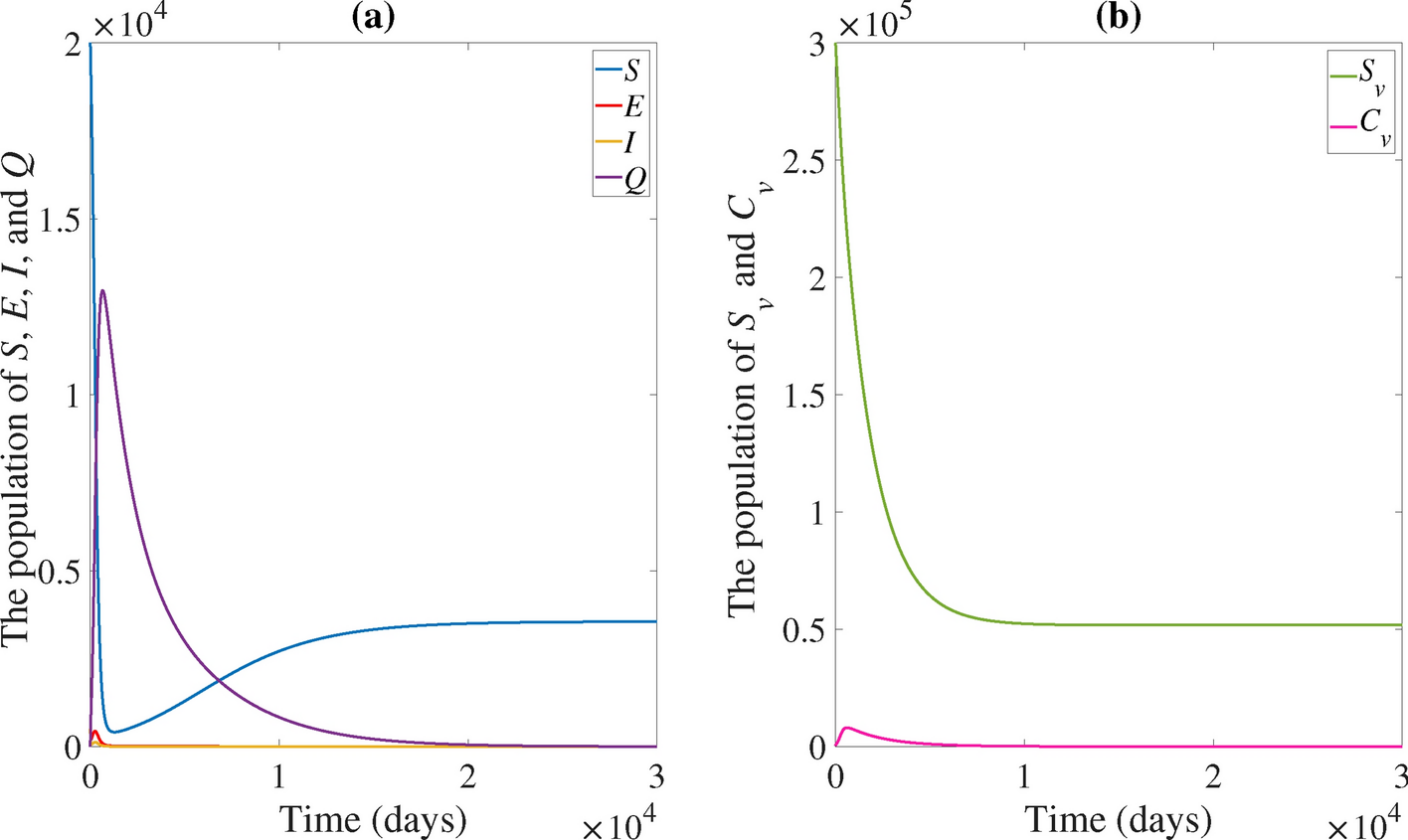
(**a**) Time series of the susceptible population (*S*), exposed population (*E*), infected population (*I*), and quarantine population (*Q*). (**b**) Time series of the susceptible vector population $(S_v)$ and contaminated vector population $(C_v)$. The solution trajectory tends toward the disease-free equilibrium (*EE*) when $R_0<1$.

**Fig. 3:**
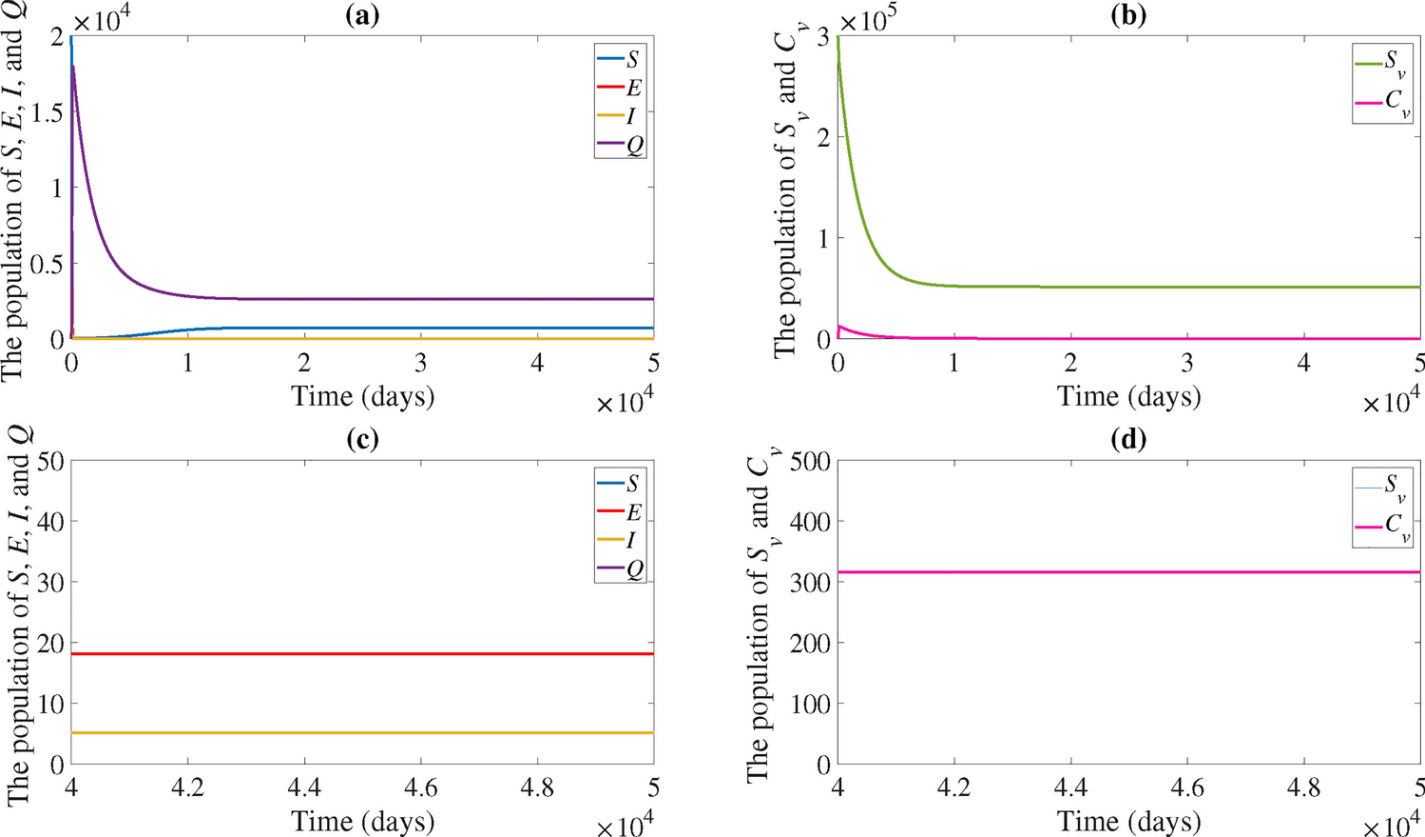
(**a**) Time series of the susceptible population (*S*), exposed population (*E*), infected population (*I*), and quarantine population (*Q*). (**b**) Time series of the susceptible vector population $(S_v)$ and contaminated vector population $(C_v)$. (**c**) Zoom-in of (a), highlighting specific trends in the population dynamics. (**d**) Zoom-in of (**b**), highlighting specific trends in the population dynamics. The solution trajectory tends toward the endemic equilibrium $(EE^*)$ when $R_0>1$.

A computer simulation of the system (3) with the parametric values settings in Table 1$(\beta _1=4.06\times 10^{-6}, \mu =5.8\times 10^{-4}, \mu _v=5.8\times 10^{-4}, \phi =0.09, \rho =0.3)$ where $R_0=0.5271<1$ is shown in Fig. 2. The solution trajectory clearly approaches to the disease-free equilibrium (*EE*) which satisfies Theorem 3.4,

If the rate of transmission from contaminated vector individuals to susceptible chicken increases by ten times (i.e. $\beta _2$ increases by ten times), the basic reproduction number will increase, resulting in $R_0 =4.8678 > 1$, as illustrated in Fig. 3. In accordance with Theorem 3.7, the solution trajectory goes to the endemic equilibrium $(EE^*)$.

### The simulation of the impulsive vaccination

**Fig. 4:**
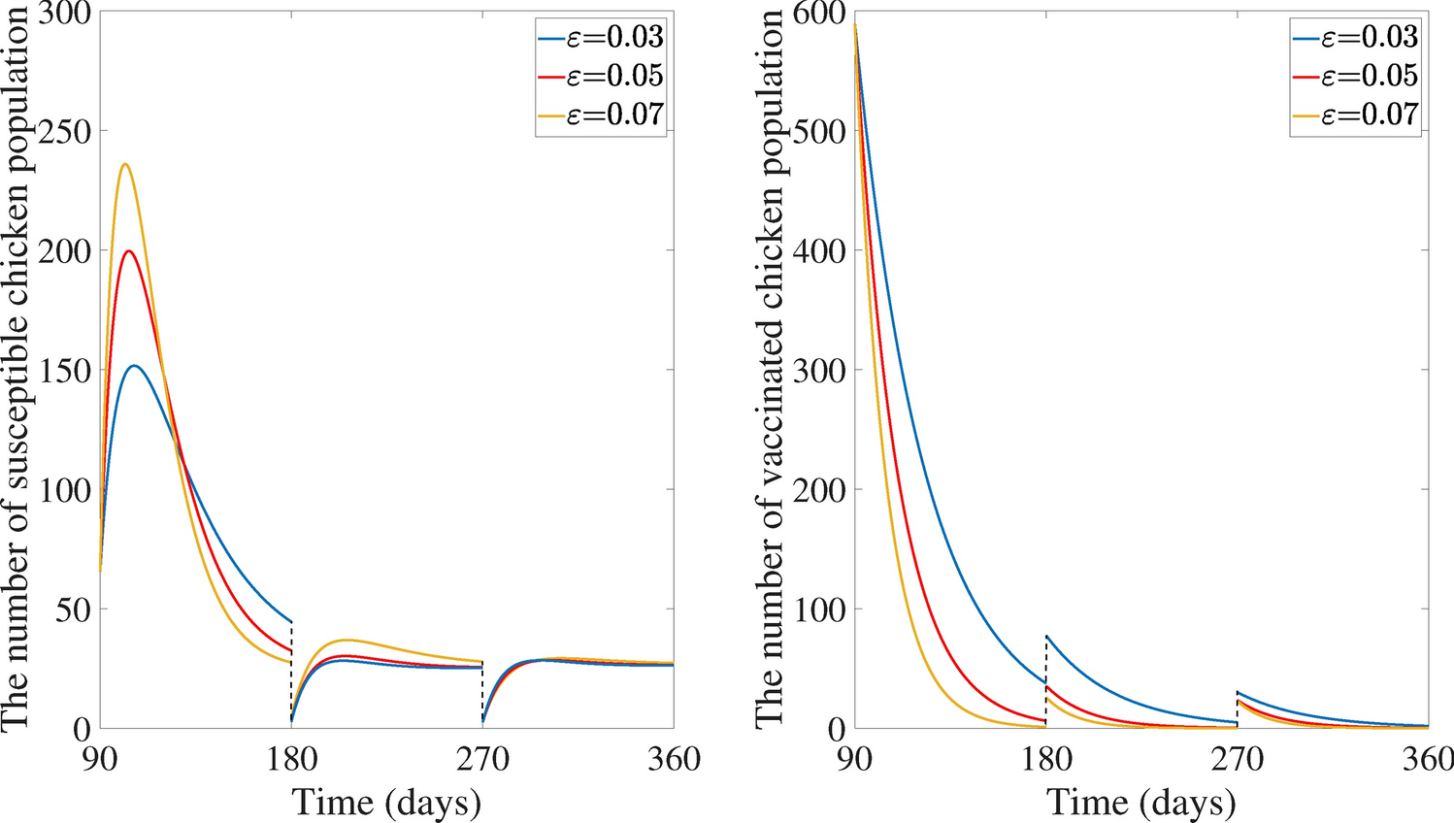
Evolution of susceptible and vaccinated populations under impulsive vaccination with $\omega = 0.9, T = 90$ days as $\varepsilon$ varies.

We simulated a scenario where a fixed proportion of the population 90% is vaccinated every 90 days $(\omega = 0.9, T = 90)$, along with varying a low vaccine immunity rate $\varepsilon$. The Fig. 4 depicts how the system behaves over time. As predicted by Theorem 4.4, the population eventually settles into a periodic pattern, where the number of susceptible and vaccinated chicken individuals repeats over time.

**Fig. 5:**
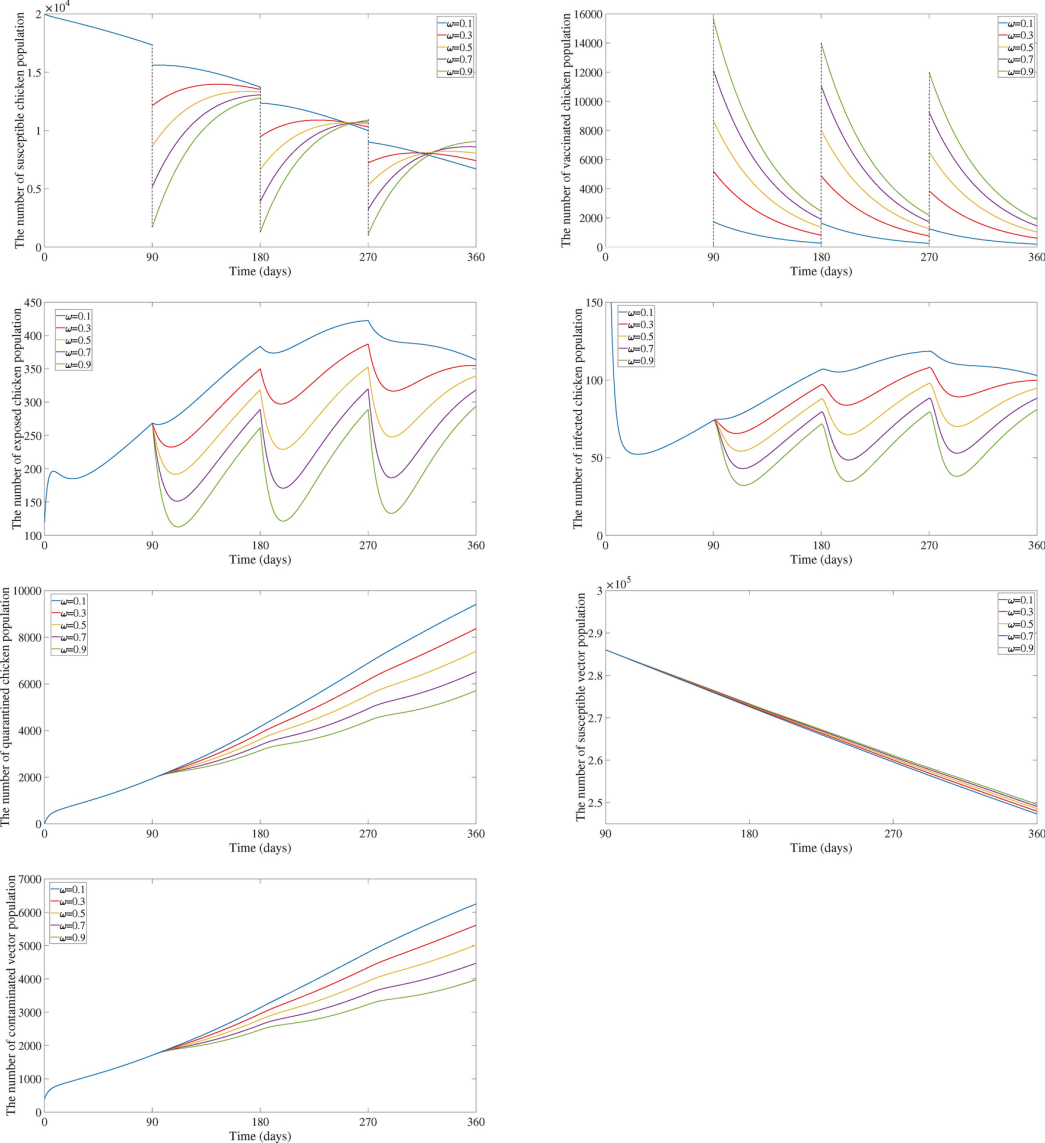
Comparison of the number of susceptible chicken, vaccinated chicken, exposed chicken, infected chicken, quarantined chicken, susceptible vector, and contaminated vector population for $T = 90$ days as $\omega$ varies.

**Fig. 6:**
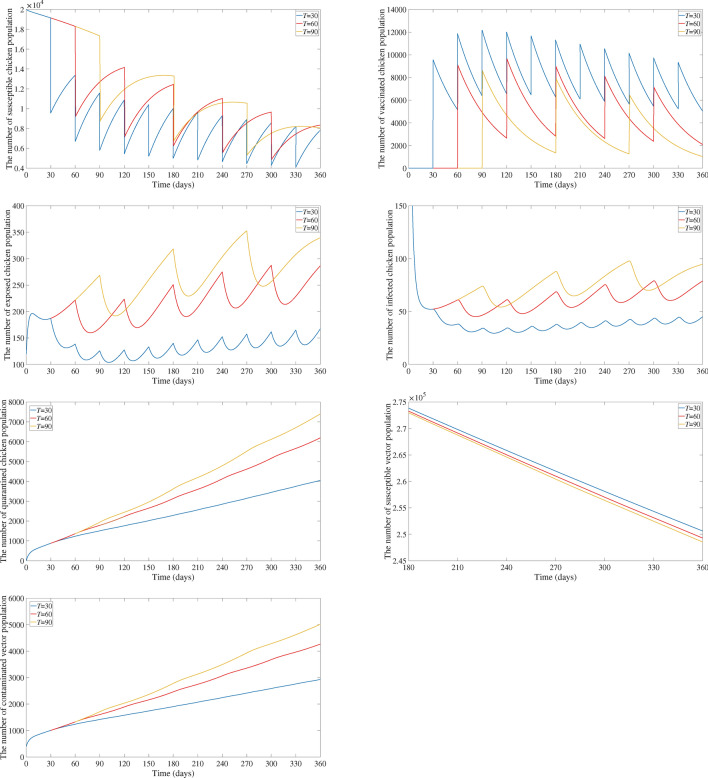
Comparison of the number of susceptible chicken, vaccinated chicken, exposed chicken, infected chicken, quarantined chicken, susceptible vector, and contaminated vector population for $\omega = 0.5$ as *T* varies.

Figures 5 and 6 display the results for $T = 90$ days as $\omega$ varies and $\omega = 0.5$ as *T* varies, respectively. It can be seen that the number of exposed chicken, infected chicken, quarantined chicken, and contaminated vector population decrease as $\omega$ increases and as *T* lowers, as would be predicted theoretically.

**Fig. 7:**
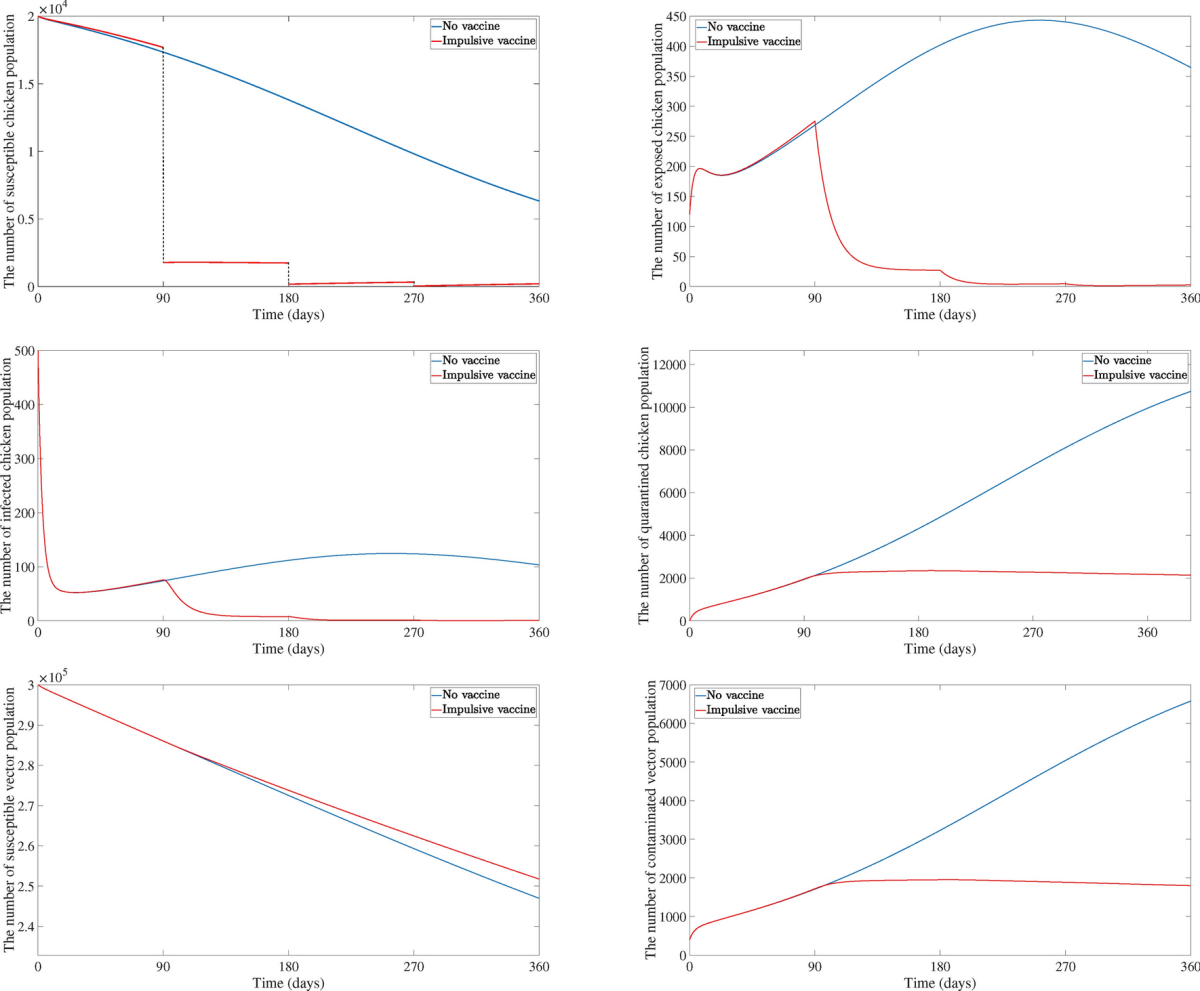
Comparison of the number of susceptible chicken, exposed chicken, infected chicken, quarantined chicken, susceptible vector, and contaminated vector population with and without impulsive vaccination ($\omega =0.9$ and $T=90$ days.).

Figure 7 compares how different populations involving the chicken farm (the susceptible chicken, exposed chicken, infected chicken, quarantined chicken, susceptible vector, and contaminated vector population) change over time with and without a program of impulsive vaccinations. The outcomes show that impulsive vaccination significantly reduces the number of exposed and infected chickens. In fact, these populations are completely eliminated within about 270 days of starting the vaccination program. On the other hand, the chicken population without impulsive vaccination spends more than 360 days to eradicate disease. However, there are some chicken population still represented in quarantine state for both with and without impulsive vaccination. The number of quarantined chicken population without impulsive vaccination increased this means that farmers must quarantine more chicken to control the disease transmission while farmers will quarantine a fixed number of chicken to control the disease spreading with impulsive vaccination.

## Conclusions and discussion

In this paper, we proposed a mathematical model for controlling the pathogen in chickens caused by vectors using vaccination and drug treatment. The model included the vector population. The insects can be considered as vectors. The population in the model can be classified into seven classes. Five classes for chicken population and two classes for vectors. These are the chicken susceptible class (*S*), the chicken vaccinated class (*V*), the chicken exposed class (*E*), the chicken infected class (*I*), the chicken quarantined class (*Q*), the vector susceptible class $(S_v)$ and the vector contaminated class $(C_v)$. The model was studied by means of theoretical ways. The basic reproduction number $R_0$ can be carried out by using the next-generation matrix method which can express the spread of the disease. The disease-free equilibrium is locally and globally asymptotically stable if $R_0<1$ as shown in Theorem 3 and Theorem 4, respectively. This means that the disease will die out if the situation satisfies this condition. Otherwise, the endemic equilibrium exists and is locally asymptotically stable if $R_0>1$. Moreover, the endemic equilibrium exists and is globally asymptotically stable if it satisfies the condition in Theorem 7. In this case, the disease will go to the endemic equilibrium level. The vaccination strategy was used to analyze the model by using impulsive behavior. The chicken will be vaccinated for a fixed period to consider the spread of the disease. The condition for the disease-free periodic solution is locally asymptotically stable if satisfy Theorem 8. This means that the disease will die out when the impulsive vaccination strategy is applied to the chicken population. The numerical simulations reveal that with impulsive vaccination the disease will die out faster than without impulsive vaccination. This is the evidence that confirm the effective of impulsive vaccination strategy. Overall this research provides the strategy to control the disease transmission in chicken. The mathematical model allows us to simulate the near future behavior of the disease transmission.

Future work on mathematical model on impulsive vaccination of vector-borne pathogen in chicken will involve:

- Modifying the model into fractional-order system of differential equations to extend the prediction capacity of the model.
- Adding some population compartment in the model which involving chicken and vectors.

## Data Availability

The datasets generated and/or analyzed in the current study are available from the corresponding author upon reasonable request.
